# What are the variables associated with Altmetric scores?

**DOI:** 10.1186/s13643-021-01735-0

**Published:** 2021-06-30

**Authors:** Amanda Costa Araujo, Adriane Aver Vanin, Dafne Port Nascimento, Gabrielle Zoldan Gonzalez, Leonardo Oliveira Pena Costa

**Affiliations:** Masters and Doctoral Programs in Physical Therapy, Universidade Cidade de São Paulo, Rua Melo Peixoto, 1407 – Tatuapé, São Paulo, SP 03070-000 Brazil

**Keywords:** Altmetric, Altmetrics, Social impact, Social media, Methodological review

## Abstract

**Background:**

Social media has been used to disseminate the contents of scientific articles. To measure the impact of this, a new tool called *Altmetric* was created. *Altmetric* aims to quantify the impact of each article through online media. This systematic review aims to describe the associations between the publishing journal and published article variables and *Altmetric* scores.

**Methods:**

Searches on MEDLINE, EMBASE, CINAHL, CENTRAL, and Cochrane Library were conducted. We extracted data related to both the publishing article and the publishing journal associated with *Altmetric* scores. The methodological quality of included articles was analyzed by the Appraisal Tool for Cross-sectional Studies.

**Results:**

A total of 19 articles were considered eligible. These articles summarized a total of 573,842 studies. Citation counts, journal impact factor, access counts, papers published as open access, and press releases generated by the publishing journal were associated with *Altmetric* scores. The magnitude of these associations ranged from weak to strong.

**Conclusion:**

Citation counts and journal impact factor are the most common variables associated with *Altmetric* scores. Other variables such as access counts, papers published in open access journals, and the use of press releases are also likely to be associated with online media attention.

**Systematic review registration:**

This review does not contain health-related outcomes. Therefore, it is not eligible for registration.

## Background

The most common way to assess the impact of an article is based on the number of citations [1]. The mean number of citations for all articles published in a journal in the preceding 2 years is called the journal’s impact factor [1]. However, the number of citations and the journal’s impact factor do not precisely reflect whether the message of the article is reaching a wider audience [2]. Currently, social media is being used to disseminate the contents of scientific articles [3, 4]. However, until recently, the impact of scientific articles on social media was not quantified. To measure this type of impact, a new score (called *Altmetric*) was created [3, 4].

**Fig. 1:**
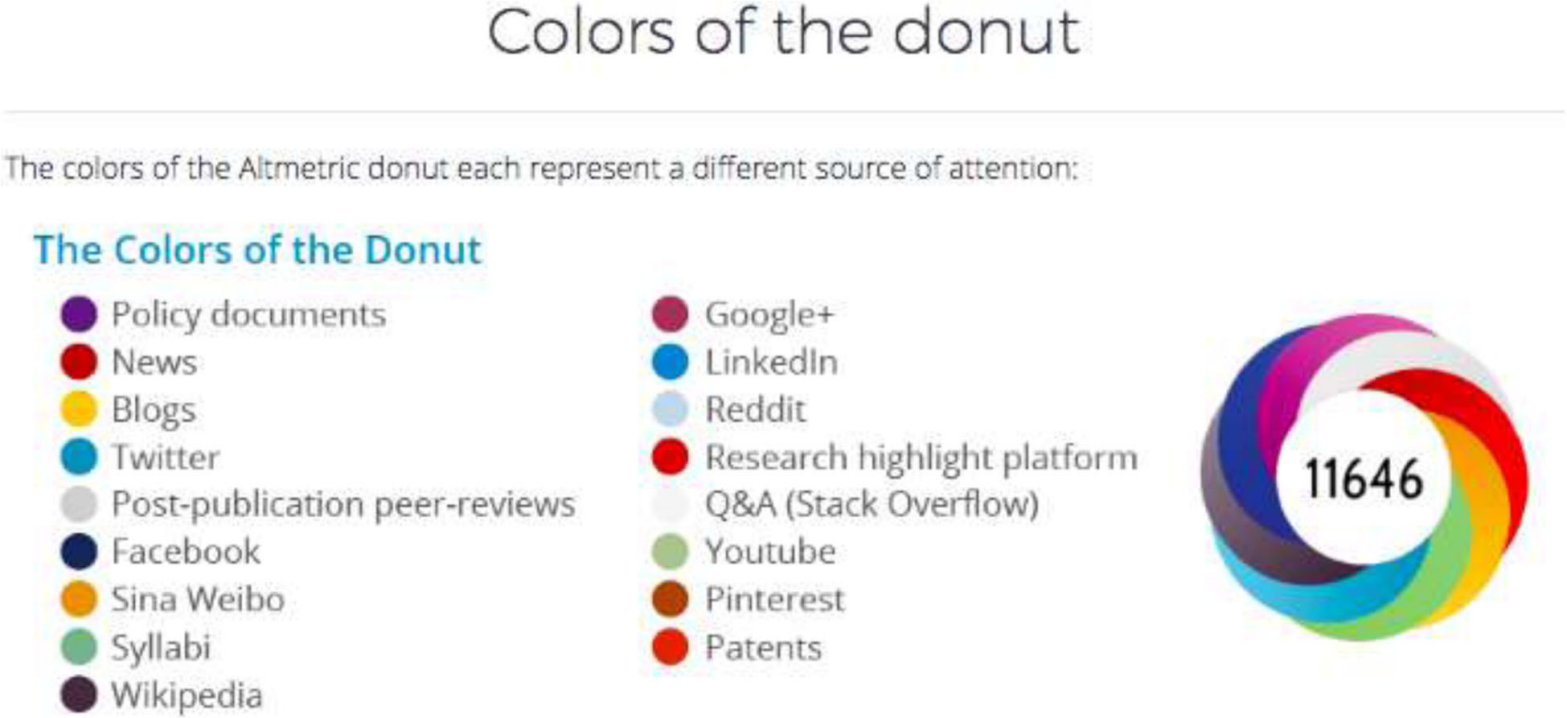
Description of the *Altmetric donut*

*Altmetric* measures the impact of each article through the attention attracted online [3]. Moreover, the *Altmetric* score reveals the instantaneous attention attracted online for articles in news outlets, comments on blogs, number of tweets, and mentions on social media. There are two types of *Altmetric* scores. The *Altmetric*-*mentioned* score includes data sources involving social media (e.g., Facebook, Twitter), newspapers, encyclopedias (e.g., Wikipedia), online platforms (e.g., Faculty1000 and publication peer reviews), videos on YouTube, question-and-answer sites (e.g., Q&A stack overflow), and policy documents in PDF form available over the internet. The *Altmetric reader* score includes data sources involving reference managers available online (e.g., Mendeley, CiteULike, and Connotea). The *Altmetric* score can be graphically represented by a “donut.” The different colors of the *Altmetric donut* represent the number of mentions on each specific online media source. For example, mentions on Twitter are represented in blue (Fig. 1).

Research about *Altmetric* has been increasing and becoming more popular in recent years [5]. However, most articles about *Altmetric* published to date are only introductory tutorials or editorials [1, 3, 4, 6, 7]. Patthi et al. [2] published a systematic review in the field of dentistry that aimed to analyze the correlations between journal citations and *Altmetric* scores. The review concluded that journal citations and *Altmetric* scores are positively correlated (with Pearson’s r ranging from 0.30 to 0.61).

Recent articles from several research fields [8, 9] showed that the number of article citations and *Altmetric* score are positively correlated. Finch et al. [10] showed that the number of tweets (i.e., an *Altmetric* component) could predict citations within the first 3 days of article publication. Araujo et al. [11] found that number of citations and journal’s impact factor were positively associated with *Altmetric* [11]. These authors also found that the number of years since publication and having a descriptive title (i.e., a title describing the aim of the study but not revealing the main conclusions) were negatively associated with *Altmetric* [11]. Therefore, it is assumed that the publishing journal and publishing article variables, such as citation counts, journal impact factor, access counts (considered the sum of HTML views and PDF downloads), papers published as open access, time since publication, and press releases generated by the publishing journal, are likely to be associated with *Altmetric* [11]. This systematic review aims to summarize all available evidence on the associations between the publishing journal and publishing article variables and *Altmetric* scores.

## Methods

### Research question

What publishing journal and publishing article variables are associated with *Altmetric* scores?

### Search strategy for identification of studies

Systematic searches were conducted on MEDLINE, EMBASE, CINAHL, CENTRAL, and Cochrane Library, as per the Cochrane Handbook [12], including publications from the inception of these databases until March 31, 2021, without language restrictions. As the topic is novel, we used only two search terms (*Altmetric* OR *Altmetrics*) in all databases to ensure a more sensitive search strategy.

### Inclusion and exclusion criteria

We included any original research studies that measured any type of association between the publishing journal and/or the publishing article with *Altmetric* scores, such as citation counts (i.e., number of citations), journal impact factor, access counts (considered the sum of HTML views and PDF downloads), papers published as open access, time since publication, and press releases generated by the publishing journal. Studies that did not have at least one of these variables were excluded. Letters to the editor, editorials, and conference abstracts were also excluded. Moreover, we excluded articles that included a subset of highly cited papers or with extremely high *Altmetric* scores.

### Data collection

Two review authors (AA and AV) independently screened all studies for eligibility and data extraction. All discrepancies identified during the stages and throughout the review were resolved via discussion or through arbitration provided by another investigator (DN). The study selection process included (1) screening the titles and abstracts and (2) screening of full-text articles.

### Data extraction

Two review authors independently extracted the following data: (1) authors, (2) year of publication, (3) research field, (4) sample size of studies analyzed, (5) study design of the included studies, (6) study aims, (7) study results, and (8) study conclusions. Variables about the publishing journal included (9) journal impact factor, (10) access counts (considered the sum of HTML views and PDF downloads), (11) papers published as open access, and (12) press releases generated by the publishing journal. Variables about the publishing articles included (13) citation counts (i.e., number of citations), and (14) time since publication. We also collected data related to (15) the *Altmetric mentioned* score and (16) the *Altmetric reader* score. We contacted authors by email to request additional information that was not reported in the original manuscripts.

### Ethics and registration

No ethical approval was required for this study. As this review has no health-related outcomes, no registration was needed [13].

### Data analysis and quality of studies

Due to a large data heterogeneity, meta-analysis was not possible. For this reason, our results are reported descriptively. The quality criteria of included articles were analyzed using an adapted version of the Appraisal Tool for Cross-sectional Studies (AXIS) [14]. This tool was developed to systematically assess the quality of cross-sectional studies by assessing 20 items. Each item is rated as “yes,” “no,” or “don’t know/no comment” [14]. The AXIS was the tool that best covered the included studies. We adapted the AXIS by excluding items 7 (*Were measures undertaken to address and categorize non-responders?*), 9 (*Were the risk factor and outcome variables measured correctly using instruments/measurements that had been trialed, piloted or published previously?*), 13 (*Does the response rate raise concerns about non-response bias?*), 14 (*If appropriate, was information about non-responders described?*), and 20 (*Was ethical approval or consent of participants attained?*), as these items are unrelated to the aims of our review.

## Results

### Search results

**Fig. 2:**
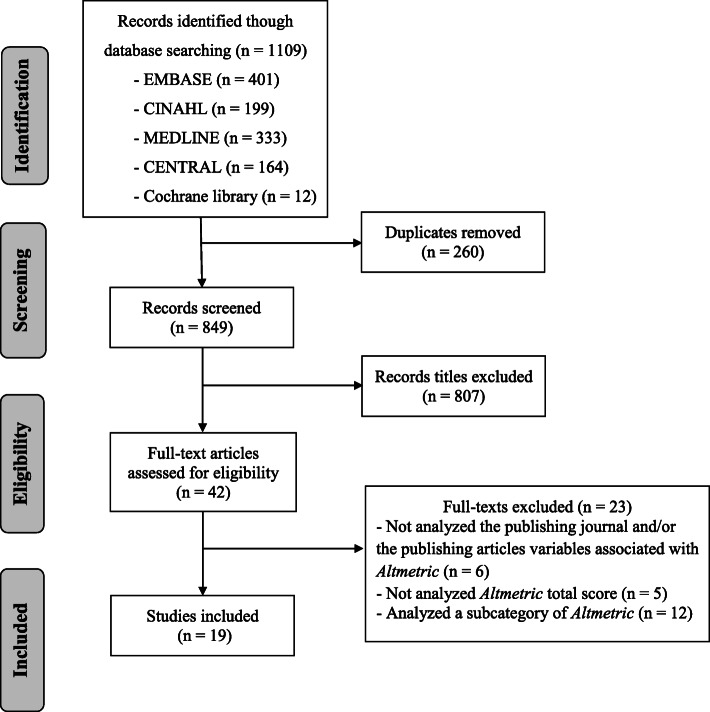
Study flow diagram of the eligibility assessment

The initial search yielded 1109 potentially eligible studies. After screening by title and abstract and removing duplicates, we considered 42 potentially eligible studies for inclusion and retrieved full-text articles. Nineteen published studies [11, 15–32] met the inclusion criteria and were included in this review. The study flow diagram of the eligibility assessment is presented in Fig. 2.

### Quality of studies

We did not consider the total score based on the instructions of the AXIS [14]. However, we observed that the studies included in general did not have good methodological quality (Table 1). We observed that most studies did not select a representative/random sample of a population (item 5), as most studies sampled the articles from main journals in their fields.

### Characteristics of included studies

The 19 eligible studies were published between 2014 and 2021 and summarized a total of 573,842 articles. The study designs of the included articles were mixed research designs [15–28, 30–32] and randomized controlled trials [11, 29]. The research fields of these articles included biomedicine [20], burn care [31], ecology and conservation [30], emergency medicine [19], engineering and technology, gastroenterology and hepatology [26], general medicine [18], joint arthroplasty [29], medical education [15], medical and natural sciences [25], multidisciplinary [22], oncology [24], physiotherapy [11, 16], plastic surgery [17], psychiatry [23], radiology [32], rheumatology [21], social sciences and humanities, solid organ transplantation [27], and spine [28]. The main objective of the included studies was to assess the association between *Altmetric* scores and variables such as citation counts (i.e., number of citations), journal impact factor, access counts (considered the sum of HTML views and PDF downloads), papers published as open access, time since publication, and/or press releases generated by the publishing journal. A summary of the methods, data analysis, results, and conclusions is presented in Table 2.

### Statistical analysis and associations of included studies

Different types of analyses were conducted in the included studies: correlation analysis [15, 17–19, 22, 23, 26–29, 31, 32], regression analysis [11, 16, 21], boosted regression trees analysis [30], principal component analysis, and factor analysis [20, 25]. The main results of the included studies demonstrated that the variables citation counts (i.e., number of citations), journal impact factor, access counts (considered the sum of HTML views and PDF downloads), papers published as open access, time since publication, and press releases generated by the publishing journal were associated with *Altmetric* scores. The magnitude of these associations ranged from weak to strong (Table 3).

**Table 1:** Quality assessment of the included studies by the AXIS tool

Articles	Introduction	Methods								Results			Discussion		Other
	1	2	3	4	5	6	8*	10	11	12	15	16	17	18	19
Amath et al. [15]	Y	Y	C^1^	Y	N	C^2^	Y	N	N	Y	Y	Y	Y	Y	Y
Araujo et al. [11]	Y	Y	C^1^	Y	Y	Y	Y	Y	Y	Y	Y	Y	Y	Y	Y
Araujo et al. [16]	Y	Y	C^1^	Y	Y	Y	Y	Y	Y	Y	Y	Y	Y	Y	Y
Asaad et al. [17]	Y	C^3^	C^1^	Y	N	Y	Y	Y	Y	Y	Y	Y	Y	Y	Y
Ayoub et al. [26]	Y	C^3^	C^1^	Y	N	C^2^	Y	N	N	Y	Y	Y	Y	Y	Y
Barakat et al. [18]	N	Y	C^1^	Y	N	C^2^	Y	N	N	Y	Y	Y	N	N	Y
Barbic et al. [19]	Y	C^3^	C^1^	Y	N	Y	Y	N	N	Y	Y	Y	Y	Y	C^4^
Bornmann et al. [20]	Y	Y	C^1^	Y	Y	Y	Y	N	Y	Y	Y	Y	Y	N	Y
Chen et al. [21]	Y	C^3^	C^1^	Y	N	C^2^	Y	Y	N	Y	Y	Y	Y	Y	Y
Costas et al. [22]	Y	C^3^	C^1^	Y	Y	Y	C^5^	N	N	Y	Y	Y	Y	Y	C^4^
Dagar et al. [23]	Y	C^3^	C^1^	Y	N	Y	Y	Y	Y	Y	Y	Y	Y	Y	Y
Didegah et al. [25]	Y	C^3^	C^1^	Y	Y	Y	N	N	Y	Y	Y	Y	Y	Y	C^4^
Haneef et al. [24]	Y	Y	C^1^	Y	Y	N	Y	Y	Y	Y	Y	Y	Y	Y	Y
Knight [27]	Y	C^3^	C^1^	Y	Y	C^2^	Y	Y	N	Y	Y	Y	Y	Y	Y
Kunze et al. [29]	Y	C^3^	C^1^	Y	N	Y	Y	Y	Y	Y	Y	Y	Y	Y	C^4^
Lamb et al. [30]	Y	C^3^	C^1^	Y	Y	Y	Y	N	Y	Y	Y	Y	Y	N	Y
Richardson et al. [28]	Y	C^3^	C^1^	Y	N	C^2^	Y	Y	Y	Y	Y	Y	Y	Y	Y
Richardson et al. [31]	Y	C^3^	C^1^	Y	N	C^2^	Y	Y	N	Y	Y	Y	Y	Y	Y
Rosenkrantz et al. [32]	Y	C^3^	C^1^	Y	N	C^2^	Y	N	N	Y	Y	Y	Y	Y	C^4^

Items 1. Were the aims/objectives of the study clear? 2. Was the study design appropriate for the stated aim(s)? 3. Was the sample size justified? 4. Was the target/reference population clearly defined? (Is it clear who the research was about?). 5. Was the sample frame taken from an appropriate population base so that it closely represented the target/reference population under investigation? 6. Was the selection process likely to select subjects/participants that were representative of the target/reference population under investigation? 7. Were measures undertaken to address and categorize non-responders? 8. Were the risk factor and outcome variables measured appropriate to the aims of the study? 9. Were the risk factor and outcome variables measured correctly using instruments/measurements that had been trialed, piloted, or published previously? 10. Is it clear what was used to determined statistical significance and/or precision estimates? (e.g., p values, CIs). 11. Were the methods (including statistical methods) sufficiently described to enable them to be repeated? 12. Were the basic data adequately described? 13. Does the response rate raise concerns about non-response bias? 14. If appropriate, was information about non-responders described? 15. Were the results internally consistent? 16. Were the results for the analyses described in the methods, presented? 17. Were the authors’ discussions and conclusions justified by the results? 18. Were the limitations of the study discussed? 19. Were there any funding sources or conflicts of interest that may affect the authors’ interpretation of the results? 20. Was ethical approval or consent of participants attained?

The items 7, 9, 13, 14, and 20 were excluded because those are unrelated to the aims of our review.

Y=Yes

N=No

C=Do not know/comment

*The risk factor analysis is not applicable for the outcome of interest of this study. Therefore, in this item, we consider only the analysis of measurement of the outcome variables

C^1^The sample size is not determined a priori because authors took in account the publications in a specific period from some journals

C^2^Authors analyzed all articles from specific journal(s), there was no description of selection process

C^3^Authors did not specify the study design

C^4^No statement about conflict of interest

C^5^The outcome variables measurement was not specified in the “Methods” section of this article

**Table 2:** Summary of the objectives and methods according to the variables of interest in the review and author’s conclusions

No	Author and year of publication (research field)	Objectives	Methods	Author’s conclusions
**1**	**Amath et al., 2017** [15] (Medical Education Journals)	To analyze the relationships among *Altmetric* score, access counts and citation counts.	**Year of search strategy:** 2012 and 2013. **Sample size:** *n* = 482. **Data extraction:** citation counts, access counts and *Altmetric* score.	*Altmetric* scores were weakly correlated with readership (access counts) and impact (citation counts).
**2**	**Araujo et al., 2018** [11] (Physiotherapy)	To analyze factors related with citation counts, journal impact factor and time since publication with *Altmetric* score.	**Year of search strategy:** between 2010 and 2015. **Sample size:** *n* = 200. **Data extraction:** citation counts, journal impact factor, time since publication and *Altmetric* score.	Researchers should preferably select high impact factor journals for submission.
**3**	**Araujo et al., 2021** [16] (Physiotherapy)	To analyze factors related with citation counts, journal impact factor, open access and time since publication with *Altmetric* score.	**Year of search strategy:** between 2015 and 2017. **Sample size:** *n* = 66 systematic reviews. **Data extraction:** citation counts, journal impact factor, open access, time since publication and *Altmetric* score.	Researchers should preferably publish their articles in journals with high impact factor (which is indirectly linked to citations).
**4**	**Assad et al., 2020** [17] (Plastic Surgery)	To analyze the relationship between citation counts and *Altmetric* score.	**Year of search strategy:** 2016. **Sample size:** *n* = 1420. **Data extraction:** citation counts and *Altmetric* score.	*Altmetric* scores were weakly correlated with citation counts.
**5**	**Ayoub et al., 2021** [26] (Gastroenterology and Hepatology)	To analyze the relationship between citation counts and *Altmetric* score.	**Year of search strategy:** 2014. **Sample size:** *n* = 4026. **Data extraction:** citation counts and *Altmetric* score.	*Altmetric* scores were strongly correlated with citation counts.
**6**	**Barakat et al., 2019** [18] (General Medicine Journals)	To analyze the relationship between citation counts and *Altmetric* score for high-impact general medicine journals.	**Year of search strategy:** 2014. **Sample size:** *n* = 551. **Data extraction:** citation counts and *Altmetric* score.	Altmetric scores were poorly correlated with the number of citations in the subsequent 3 years.
**7**	**Barbic et al., 2016** [19] (Emergency Medicine)	To analyze the citation counts, journal impact factor and *Altmetric* scores in emergency medicine journals.	**Year of search strategy:** 2014. **Sample size:** *n* = 50. **Data extraction:** citation counts, journal impact factor and *Altmetric* score.	*Altmetric* scores were weakly correlated with citation counts and journal impact factor.
**8**	**Bornmann et al., 2018** [20] (Biomedical Area)	To analyze the dimensions of measurement for citation counts and *Altmetric*.	**Year of search strategy:** between 2011 and 2013. **Sample size:** *n* = 33.683. **Data extraction:** citation counts and *Altmetric* score.	*Altmetric reader* score are associated to citation counts.
**9**	**Chen et al., 2019** [21] (Rheumatology)	To analyze the relationship between citation counts and *Altmetric* score.	**Year of search strategy:** between 2010 and 2015. **Sample size:** *n* = 1460. **Data extraction:** citation counts and *Altmetric* score.	Disease area did not correlate with *Altmetric* and citations counts. *Altmetric* identified different articles as high impact compared with citation metrics.
**10**	**Costas et al., 2015** [22] (Multidisciplinary)	To analyze the relationship between citation counts, journal impact factor and *Altmetric* score.	**Year of search strategy:** 2013. **Sample size:** *n* = 500.229. **Data extraction:** citation counts, journal impact factor and *Altmetric* score.	*Altmetric* scores were weakly correlated with citations. This findings suggests that the potential of *Altmetric* to replace traditional citation analysis is not very strong.
**11**	**Dagar et al., 2021** [23] (Psychiatry)	To analyze the relationship between citation counts and *Altmetric* score.	**Year of search strategy:** 2016. **Sample size:** *n* = 360. **Data extraction:** citation counts and *Altmetric* score.	*Altmetric* scores were weakly correlated with citation counts. Besides that, the authors found a very high degree of public engagement with psychiatry research.
**12**	**Didegah et al., 2018** [25] (Social Sciences & Humanities, Engineering & Technology, Medical & Natural Sciences)	To analyze the differences between citation counts and *Altmetric* score.	**Year of search strategy:** between 2012 and 2014. **Sample size:** *n* = 13.623. **Data extraction:** citation counts and *Altmetric* score.	*Altmetric reader* score are associated to citation counts.
**13**	**Haneef et al., 2017** [24] (Oncology)	To analyze the variables journal impact factor, press release and open access with *Altmetric* score of articles evaluating cancer treatments.	**Year of search strategy:** first 6 months of 2014. **Sample size:** *n* = 792. **Data extraction:** journal impact factor, press release, open access and *Altmetric* score.	The press release and the journal impact factor are the most important factors associated with high online media attention were the presence.
**14**	**Knight, 2014** [27] (Solid Organ Transplantation)	To analyze the association between citation counts and *Altmetric* score in the field of solid organ transplantation.	**Year of search strategy:** between August 2011 and July 2012. **Sample size:** *n* = 6.979. **Data extraction:** citation counts and *Altmetric* score.	*Altmetric* scores were weakly correlated with citation counts. Blogging and expert recommendation, in particular, are associated with higher citation rates.
**15**	**Kunze et al., 2020** [29] (Joint Arthroplasty)	To analyze the relationship between citation counts and the *Altmetric* score.	**Year of search strategy:** 2016. **Sample size:** *n* = 42. **Data extraction:** citation counts and *Altmetric* score.	High methodologic quality and limited study bias markedly contribute to the *Altmetric* of RCTs in the total joint arthroplasty literature.
**16**	**Lamb et al., 2018** [30] (Ecology and Conservation)	To analyze the association between citation counts and the *Altmetric* score.	**Year of search strategy:** between 2005 and 2015. **Sample size:** *n* = 8.322. **Data extraction:** citation counts and *Altmetric* score.	There are strong association between science communication (measured by the *Altmetric* score) and citation counts.
**17**	**Richardson et al., 2020** [28] (Spine Journals)	To analyze the relationship between citation counts and the *Altmetric* score.	**Year of search strategy:** January, February and March 2017. **Sample size:** *n* = 380. **Data extraction:** citation counts and *Altmetric* score.	*Altmetric* scores were weakly correlated with citation counts in seven spine journals.
**18**	**Richardson et al., 2021** [31] (Journal of Burn Care & Research and Burns)	To analyze the relationship between citation counts and the *Altmetric* score.	**Year of search strategy:** 2017. **Sample size:** *n* = 285. **Data extraction:** citation counts and *Altmetric* score.	*Altmetric* scores were weakly correlated with citation counts. Besides that, the authors recommend the combined use of *Altmetric* and traditional metrics such as citation count and impact factor.
**19**	**Rosenkrantz et al., 2017** [32] (Radiology)	To analyze citation counts and *Altmetric* score for articles in popular general radiology journals.	**Year of search strategy:** 2013. **Sample size:** *n* = 892. **Data extraction:** citation counts and *Altmetric* score.	Articles published in four popular radiology journals overall received relatively low attention on social media comparison with citations.

Summary of the association between variables of the publishing

**Table 3:** Summary of the association between variables of the publishing journal and the publishing articles with *Altmetric* scores

Studies and analyses	Citation counts	Journal impact factor	Access counts	Open access	Time since publication	Press release
**Correlation analysis**						
Amath et al. [15]	r = 0.25		r = 0.30			
Assad et al. [17]	r = 0.33					
Ayoub et al. [26]	r = 0.62					
Barakat et al. [18]	r = 0.33					
Barbic et al. [19]	r = 0.22	r = 0.35				
Costas et al. [22]	r = 0.18	r = 0.19				
Dagar et al. [23]	r = 0.43					
Knight [27]	r = 0.16* and r = 0.23**					
Kunze et al. [29]	r = 0.36					
Richardson et al. [28]	r = 0.32					
Richardson et al. [31]	r = 0.12					
Rosenkrantz et al. [32]	r = 0.20					
**Linear regression analysis**						
Chen et al. [21]	R^2^ = 0.00					
**Multivariate regression analysis**						
Araujo et al. [11]	β = 5.2* and β = 10.1**	β = 3.4			β = −4.9	
Araujo et al. [16]	β = 2.9* and β = 6.37**	β = 15.36* and β = −3.21**		β = 0.74* and β = 4.04**	β = −21.99* and β = 18.13**	
Haneef et al. [24]		RoM = 1.10		RoM = 1.48		RoM = 10.14
**Boosted regression trees analysis**						
Lamb et al. [30] *Altmetric* scores were strongly associated with citation counts.						
**Principal component analysis and factor analysis**						
Bornmann et al. [20] *Altmetric reader* score are associated to citation counts.						
Didegah et al. [25] *Altmetric reader* score are associated to citation counts.						

*These numbers represent *Altmetric mentioned*. **These numbers represent *Altmetric reader*. r = correlation estimates. RoM = regression coefficients represent the logarithm of ratio of mean. β = β coefficient

## Discussion

We aimed to summarize all available evidence on the associations between the publishing journal and publishing article variables and *Altmetric* scores. We found that citation counts (i.e., number of citations), journal impact factor, access counts (considered the sum of HTML views and PDF downloads), papers published as open access, time since publication, and press releases generated by the publishing journal were associated with *Altmetric* scores. The magnitude of these associations ranged from weak to strong. In addition, we observed that citation counts and journal impact factor were associated with *Altmetric* scores in all included studies [11, 15–32].

There is a previous systematic review about the correlation between citation counts and *Altmetric* in medical research [2]. Moreover, there are articles that have measured associations between citation counts and *Altmetric* scores [15, 32]. In accordance with the systematic review [2] and these articles [15, 32], we found a positive correlation between citation counts and *Altmetric* scores. Similarly, our overview indicated positive associations (ranging from weak to moderate) between citation counts and *Altmetric* scores [11, 15–32]. These results are similar to those related to a journal’s impact factor [11]. This is not surprising, because a journal’s impact factor is based on citation counts of scientific articles [1]. We also found that most included studies, with the exception of those by Araujo et al. [11, 16] and Knight [27], did not analyze the *Altmetric reader* score. Thus, our findings are largely based on the *Altmetric-mentioned* score. We strongly recommend that further investigations on *Altmetric reader* score be conducted.

Regarding the quality of studies, the main limitation we observed was the lack of reporting the methods in detail. Items related to sampling, selection criteria, and statistical analysis in general were poorly described. On the other hand, the articles were clear in terms of data analysis and results. Finally, most authors presented the limitations of the study in their discussion and disclosed their potential conflicts of interest.

No studies identified specific characteristics of articles, for example, analysis of studies that published popular/hot topics (e.g., studies on coronavirus, miraculous diets, cancer prevention, early life on earth, religious evidence). Moreover, there is no analysis of studies comparing whether the direction of the results (i.e., positive versus negative conclusions) influences *Altmetric* scores. These characteristics are likely to increase the number of people who access and share these articles on social media [11]. We recommend that future studies identify if these characteristics are associated with *Altmetric* scores.

Finally, we propose 4 suggestions to improve the social impact and visibility of scientific articles: (1) select high impact factor journals for submission of articles; (2) use provocative titles (titles expressing the results of the trial) or interrogative titles; (3) use social media (Twitter, Facebook, etc.), websites, and blogs to disseminate principal findings; and (4) post the article with its digital object identifier (DOI) or the journal’s link to the article to be captured by *Altmetric*. These simple strategies are likely to improve the visibility of articles to a larger readership [5, 33]. The major strength of this study is the inclusion of articles from all fields of the research (*n* = 565,352 articles analyzed). On the other hand, a possible limitation of this study is the large heterogeneity of the included studies. Because of this, the data were analyzed only descriptively. Another potential limitation of our review is related to the selection of the databases we chose. We decided to cover the most comprehensive databases, such as MEDLINE, CINAHL, EMBASE, Cochrane Library, and CENTRAL, and we might have missed some eligible articles published in smaller databases or gray literature.

## Conclusion

Citation counts, journal impact factor, access counts (considered the sum of HTML views and PDF downloads), papers published as open access, time since publication, and press releases generated by the publishing journal were associated with *Altmetric* scores.

## Data Availability

The datasets used and/or analyzed during the current study are available from the corresponding author on reasonable request.
